# What is the difference between missing completely at random and missing at random?

**DOI:** 10.1093/ije/dyu080

**Published:** 2014-04-04

**Authors:** Krishnan Bhaskaran, Liam Smeeth

**Affiliations:** Department of Non-communicable Diseases Epidemiology, London School of Hygiene and Tropical Medicine, London, UK

**Keywords:** missing data, missing at random, multiple imputation

## Abstract

The terminology describing missingness mechanisms is confusing. In particular the meaning of ‘missing at random’ is often misunderstood, leading researchers faced with missing data problems away from multiple imputation, a method with considerable advantages. The purpose of this article is to clarify how ‘missing at random’ differs from ‘missing completely at random’ via an imagined dialogue between a clinical researcher and statistician.

**Key Messages**

- The terms ‘missing at random’ and ‘missing completely at random’ are used to describe assumptions about missing data that are needed for standard implementations of multiple imputation, but the meanings of these terms are often confused.
- When observations of a variable are missing completely at random, the missing observations are a random subset of all observations; the missing and observed values will have similar distributions.
- Missing at random means there might be systematic differences between the missing and observed values, but these can be entirely explained by other observed variables.
- For example, if blood pressure data are missing at random, conditional on age and sex, then the distributions of missing and observed blood pressures will be similar among people of the same age and sex (e.g. within age/sex strata).

## Introduction

Missingness in a dataset can be categorised as ‘missing completely at random’, ‘missing at random’ and ‘missing not at random’.^1^ Under the assumption of ‘missing at random’ or ‘missing completely at random’, standard implementations of multiple imputation methodology can be used; this has substantial advantages, as it allows missing data to be handled in a way that is unbiased and statistically valid.^1–4^ However, the terminology describing missingness mechanisms is undeniably confusing. In particular, ‘missing at random’ is often conflated with ‘missing completely at random’, leading researchers to mistakenly conclude that any systematic patterns or mechanisms underlying the missing data contraindicate the use of multiple imputation. In this article, an imagined dialogue between a clinical researcher and statistician is presented. This aims to clarify the real meaning of ‘missing at random’, and to demonstrate how one can think through whether the assumption is likely to be met in real clinical contexts.

## Dialogue

Clinical researcher: I'm not considering multiple imputation for my study because I read that the data have to be missing at random. I am using routine health records, and I have missing blood pressure data. But they won't be randomly missing, because people with blood pressure missing are totally different to people with blood pressure recorded.

Statistician: What do you mean by different?

Clinical researcher: Older people or people with cardiovascular disease are more likely to have their blood pressure measured and recorded as part of their care, and young healthy people are more likely to have blood pressure missing. But the former are precisely the people whose blood pressures are likely to be higher, whereas the latter will tend to have lower (healthy) blood pressures. So, it follows that the people with blood pressure missing are likely to have lower blood pressures on average than those with blood pressure recorded. Blood pressure is clearly not randomly missing.

**Figure 1.**
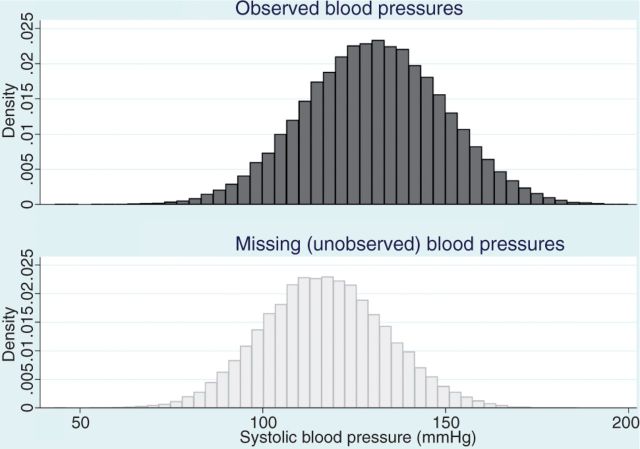
Distribution of systolic blood pressure (simulated data) comparing those with blood pressure recorded (top panel) and those with blood pressure missing (bottom panel)—blood pressure is missing at random conditional on age and cardiovascular disease. Simulated data with 100 000 observations, divided into two age groups (young, elderly) and with a randomly assigned binary cardiovascular disease (CVD) variable. Among those with no CVD, mean systolic blood pressure (SBP) was set at 110 mmHg in the young age group, 120 mmHg in the elderly. Mean SPB was set 15 mmHg higher where CVD was present. Individual normally distributed observations were simulated with standard deviation 15 mmHg. The probability of SBP being missing was 0.8 in the young age group with no CVD, 0.4 in the young age group with CVD, 0.2 in the elderly with no CVD and 0.1 in the elderly with CVD

Statistician: That doesn't mean your blood pressure data can't be ‘missing at random’. Let’s take a step back. You are worried that the distribution of blood pressures in the patients with missing data would look different, if you could observe it, to the distribution of blood pressures in the patients with complete data. An imaginary histogram of the missing blood pressures would show a distribution shifted towards lower blood pressures, compared with your histogram of the observed blood pressures (see Figure 1)?

Clinical researcher: Exactly.

Statistician: I agree. But that only tells us that blood pressure is not ‘missing completely at random’. If blood pressure were ‘missing completely at random’, those histograms would look the same: the distribution of missing and observed blood pressures would be similar.

Clinical researcher: So what is the difference between ‘missing completely at random’ and ‘missing at random’?

Statistician: They are quite different assumptions. Admittedly the terminology is not particularly helpful. ‘Missing completely at random’ means what it says: the observations with missing blood pressure are just a random subset of all observations, so there are no systematic differences between the missing and observed blood pressures. ‘Missing at random’ means that there might be systematic differences between the missing and observed blood pressures, but these can be entirely explained by other observed variables. Tell me again what you believe to be the main factors that would drive differences between the missing and observed blood pressures in your data?

Clinical researcher: Age and cardiovascular disease.

Statistician: Right. You argued that young patients who have no cardiovascular disease will tend to have blood pressure missing, and will also tend to have lower blood pressure than those who are older and/or have cardiovascular disease. So the systematic differences in blood pressure between the patients with missing data and those with complete data are, at least to some extent, explained by differences in age and cardiovascular disease between these two groups. Now, do you know the age of the people in your dataset?

Clinical researcher: Of course.

Statistician: And whether they have any cardiovascular disease?

Clinical researcher: Yes, we have reasonable information on that.

Statistician: Great—then you might still be able to assume blood pressure is ‘missing at random’! Think about groups of people that are similar in terms of age and cardiovascular disease. For example, you could imagine dividing the patients in your study into people in their 20 s with no cardiovascular disease, people in their 20 s with cardiovascular disease, people in their 30 s with no cardiovascular disease, people in their 30 s with cardiovascular disease, and so on.

Clinical researcher: You mean stratify by age and cardiovascular disease?

Statistician: Exactly; in your mind, at least. In statistical terms we are constructing an argument that ‘conditions’ on age and cardiovascular disease (Box 1). Mentally stratifying your dataset into subgroups based on age and cardiovascular disease is one way to picture this. Now, take one stratum: for example, people in their 20s with no cardiovascular disease. In that group, there may be some people with blood pressure complete and some people with blood pressure missing?

**Box 1. Conditional statements**

When statisticians make a statement that is ‘conditional’, this can usually be translated into a simple caveat or ‘if’ clause. Conditioning on covariates just means that we restrict our thinking to people that are similar in terms of those covariates. In the present example, the statement that blood pressure is missing at random conditional on age and cardiovascular disease can be translated as: IF we restrict to any group that is similar in terms of age and cardiovascular disease, then blood pressure is missing (completely) at random.

Clinical researcher: Yes.

Statistician: Within this group of people that are uniformly young and free of cardiovascular disease, do you still believe that the people with missing blood pressure data have systematically different blood pressure to those with complete blood pressure data?

Clinical researcher: I'm not sure. Admittedly, it's not as easy to think why they would be systematically different within that particular group.

**Figure 2.**
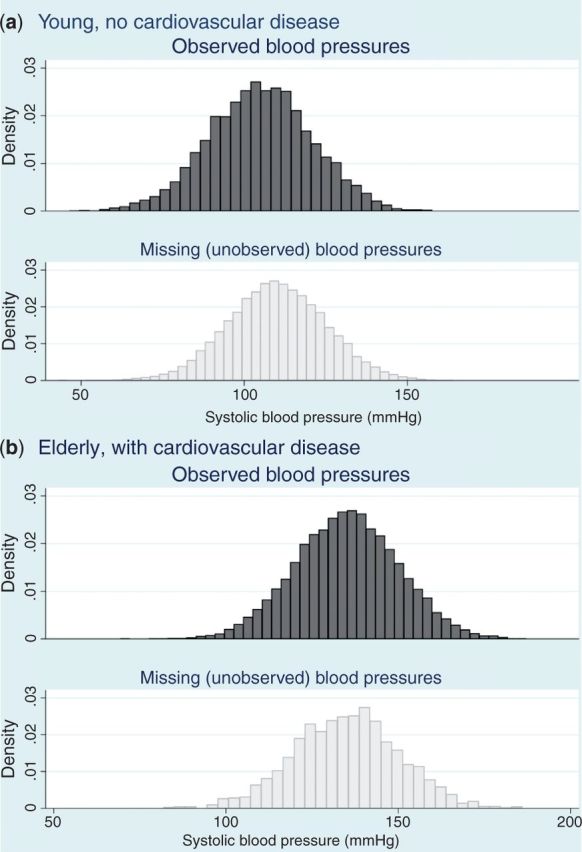
Distribution of systolic blood pressure comparing those with blood pressure recorded and those with blood pressure missing, within age/cardiovascular disease strata (simulated data) –—blood pressure is missing at random conditional on age and cardiovascular disease. Generated from the same simulated dataset as described in the footnote to Figure 1

Statistician: Now extend that thinking to all of your age/cardiovascular disease strata. If you can convince yourself that within each stratum, the distribution of missing blood pressures is likely to be similar to the distribution of observed blood pressures, then your blood pressure data can be assumed to be ‘missing at random’, conditional on age and cardiovascular disease (see Figure 2).

Clinical researcher: I understand. But I just thought of something: even in the young healthy group, there might be differences. I suspect that men are more likely to have blood pressure missing because on average they make fewer visits to the doctor, but there are data suggesting men are more likely to have higher blood pressure. That means that even among young people with no cardiovascular disease, those with missing data will more likely be men, and if men tend to have higher blood pressure, the distribution of blood pressures among those with missing data will still be shifted upwards, compared with those with complete data. Missing not at random!

Statistician: But you have data on gender: you don't need to limit yourself to age and cardiovascular disease. You can mentally stratify your data further, on gender, pregnancy, other morbidities; any other relevant variables that you have data on. Then, if you want to argue that your data are missing not at random, you will need to convince yourself that even among a group of people who are the same in terms of all of your recorded variables, like age, cardiovascular disease status, gender, pregnancy status, other morbidities and so on, there are still systematic differences in the blood pressure distribution between those with missing and complete blood pressure data.

Clinical researcher: In other words, I need to have to think carefully about all the information I have available in the dataset before I can produce a convincing argument one way or the other?

Statistician: Quite! Often, researchers discount the idea of using multiple imputation, because they misunderstand ‘missing at random’ to mean, well, to mean what it says! In fact it is a weaker assumption than ‘missing completely at random’. ‘Missing at random’ is by no means likely to be satisfied in every study, but whether the assumption is reasonable deserves to be considered carefully, because multiple imputation has considerable advantages. If the underlying assumptions are met, it allows missing data to be accounted for in a statistically valid and unbiased way.

Clinical researcher: Can’t I just test whether the data are missing at random, using the dataset?

Statistician: Impossible! It is a fundamentally untestable assumption, because it concerns the unobserved values. For example, in your dataset, within each of your strata, you would need to know the distribution of blood pressure among people with no blood pressure recorded, in order to compare it with the distribution of blood pressure among those with complete data. So it really is an assumption that you need to justify based on background knowledge and discussion with experts.

Clinical researcher: In what circumstances do you think it's really hard for a variable to satisfy the ‘missing at random’ assumption?

Statistician: It depends on the variable, and on the context. But sometimes the recording of a particular variable is likely to fundamentally depend on the value of that variable, even among groups of patients that are similar in other ways. It's easy to imagine this happening for parameters that are quite visible to a clinician, and which can vary between otherwise similar patients. An example would be a study using body mass index (BMI) data from routine clinical care, such as from a primary care health records database. Routinely collected health data of this kind are increasingly used for research, but parameters like BMI are unlikely to be complete for all patients in the database, so we need to think carefully about whether the ‘missing at random’ assumption is likely to be reasonable. Imagine a group of patients that are very similar in terms of demographics and recorded medical history, but some are overweight and some are healthy weight; you could easily imagine that BMI is more likely to be recorded for an overweight patient, because the clinician will see that the patient is overweight and will consider it clinically relevant and worthy of measuring and recording. Then, even within narrow strata defined by all the available data we have on the patients, the distribution of BMI will still tend towards lower BMIs for those with missing BMI data, and higher BMIs for those with complete BMI data. In that kind of situation, there is a quite convincing argument that the data really are likely to be ‘missing not at random’, and multiple imputation would not be valid.

## Funding

No specific funding was received for this work. K.B. is supported by a National Institute for Health Research postdoctoral fellowship (PDF-2011-04-007). L.S. is supported by a senior Wellcome fellowship in clinical science (grant number 082178).

**Conflict of interest:** None declared.

## References

[1] Sterne JA White IR Carlin JB Multiple imputation for missing data in epidemiological and clinical research: potential and pitfalls. BMJ 2009;338:b2393. 10.1136/bmj.b2393 PMC2714692 19564179

[2] Carpenter J Kenward M Multiple Imputation and its Application. Chichester, UK: John Wiley & Sons, 2012.

[3] Rubin DB Multiple Imputation for Nonresponse in Surveys. New York: Wiley, 1987.

[4] Greenland S Finkle WD A critical look at methods for handling missing covariates in epidemiologic regression analyses. Am J Epidemiol 1995;142:1255–64. 7503045 10.1093/oxfordjournals.aje.a117592

